# The influence of statin exposure on inflammatory markers in patients with early bacterial infection: pilot prospective cohort study

**DOI:** 10.1186/1471-2253-14-106

**Published:** 2014-11-19

**Authors:** Manu Shankar-Hari, Antonia Donnelly, Ruxandra Pinto, Zaid Salih, Cathrine McKenzie, Marius Terblanche, Neill KJ Adhikari

**Affiliations:** Division of Asthma, Allergy and Lung Biology, King’s College London, Department of Critical Care Medicine, St. Thomas’ Hospital, 1st Floor, East Wing, Westminster Bridge Road, London, SE1 7EH UK; Emergency Department, University Hospital Southampton NHS Foundation Trust, Tremona Road, Southampton, SO16 6YD UK; Department of Critical Care Medicine, Sunnybrook Health Sciences Centre, 2075 Bayview Avenue, Toronto, M4N 3M5 Canada; School of Pharmacy, University College London, London, UK; Department of Pharmacy and Critical Care, Guys and St Thomas NHS Foundation Trust, St Thomas’ Hospital, Lambeth Palace Road, London, SE1 7EH UK; Institute of Pharmaceutical Sciences, Kings College London, London, UK; Centre for Critical Illness Data Sciences Research, Division of Health & Social Care Research, King’s College London, Department of Critical Care Medicine, 1st Floor, East Wing, St. Thomas’ Hospital, Westminster Bridge Road, London, SE1 7EH UK; Department of Critical Care Medicine, Sunnybrook Health Sciences Centre, Sunnybrook Research Institute and University of Toronto, 2075 Bayview Avenue, Toronto, M4N 3M5 Canada

**Keywords:** Sepsis, Biomarkers, Surrogate end-points, Progression of illness

## Abstract

**Background:**

In the context of infection, progressive illness resulting in acute organ dysfunction is thought to be secondary to inflammatory response. Our aims were to determine risk factors for progressive illness following infection in a low-risk hospitalised cohort, including the impact of prior stain therapy.

**Methods:**

We performed a prospective observational cohort study on two adult acute medical wards of a single tertiary academic hospital. We screened drug prescription charts of all adult acute medical admissions for inclusion criteria of inpatient administration of antibiotics for more than 24 hours for a microbiologically confirmed or clinically suspected infection. Patients were followed until admission to a high dependency unit (HDU) or intensive care unit (ICU), discharge from hospital, or to a maximum of 10 days. Outcomes were evolution of systemic inflammatory response syndrome (SIRS) criteria, white cell count and C-reactive protein measurements, and adverse clinical outcomes. We constructed multivariable models accounting for repeated within-patient measurements to determine associations between a priori selected predictors (days since admission, age, gender, Charlson score, prior statin exposure) and selected outcomes.

**Results:**

We enrolled 209 patients; 27.8% were statin users and the commonest infection was pneumonia (51.0%). Most (88.0%) had at least 1 SIRS criterion on admission, and 76 (37.3%) manifested additional SIRS criteria over time. Risks of admission to HDU/ICU (3.3%) and of 30-day mortality (5.7%) were low. The proportion of patients with at least 1 SIRS criterion, mean CRP, and mean WBC all decreased over time. Multivariable regression models identified days since hospital admission as the only variable associated with daily presence of SIRS criteria, WCC, or CRP (adjusted OR <1 and p < 0.0001 in all analyses). Statin exposure was not a significant predictor.

**Conclusions:**

This cohort of ward patients treated for infection had a low risk of clinical deterioration, inflammatory markers improved over time, and statin exposure was not associated with any outcome. Future larger studies may identify risk factors for progression of illness in this population and plausible surrogate endpoints for evaluation in clinical trials.

## Background

Systemic inflammatory response in the presence of infection defines sepsis [1]. Patients admitted acutely to hospital with infections are at risk of developing a progressive host inflammatory response leading to organ dysfunction [2–4], a group of heterogeneous syndromes defined as severe sepsis [1]. Patients managed in an intensive care unit (ICU) with sepsis are also at risk developing a progressive illness trajectory associated with adverse outcomes [3, 5, 6]. The ICU-treated population incidence of sepsis is substantial (1 ± 0.5 cases per 1000) [7]. Given this risk of organ dysfunction [8, 9], the reported minimal treatment effects and potential for harm with interventions for established organ failure [10, 11], an alternative approach is to evaluate drugs with pleotropic effects to prevent progressive organ dysfunction in a pre-ICU population of patients with infection.

One candidate drug class to prevent organ dysfunction in at-risk patients are statins, for which biological plausibility and proof of concept data exist for salutary effects on infection-related inflammatory response [12, 13]. However, to design a randomized controlled trial (RCT) of any intervention to prevent organ dysfunction in patients with acute infection, it is crucial to understand the incidence, timing, and risk factors for acute organ dysfunction [14]. We therefore conducted a prospective cohort study of patients hospitalised with infection to determine the evolution of clinical and laboratory criteria for the systemic inflammatory response syndrome (SIRS) variables, and secondarily, the impact of prior statin therapy on this outcome.

## Methods

### Study design and setting

We performed a prospective observational cohort study on two adult acute medical wards of a single tertiary academic hospital, to which non-surgical patients with infections are routinely admitted. These patients are reviewed twice daily in post-take rounds led by a senior clinician. The use of antibiotics, including selection, start time, and duration is managed by the treating team with input from pharmacists and daily microbiology rounds. The study was approved by the Research Ethics Committee of Guy’s & St Thomas’ NHS Foundation Trust. Given that only anonymised data extracted from clinical records were used, the Research Ethics Committee waived the need for informed consent.

### Participants

We screened the drug prescription chart of all patients admitted between April and September 2008 and selected patients >16 years old with a working or confirmed diagnosis of infection, defined by administration of antibiotics for more than 24 hours since admission for a microbiologically confirmed or clinically suspected infection. We excluded those who had received prophylactic antibiotics; antibiotics received for less than 24 hours; and direct admissions to a high dependency unit (HDU) or ICU.

### Data collection and follow-up

All study variables were obtained from paper medical records and the hospital’s clinical information system. For all enrolled patients we prospectively collected baseline data, including demographics, co-morbidities (using the Charlson Index [15]), chronic medications including prior statin therapy, and details of current illness (type of infection, severity of illness). To determine statin exposure, we used information obtained from clinical records to determine whether a patient took a statin for most days of the 4 weeks before admission. Accurate medication history is routinely collected by ward-based pharmacists who participate in post-admission ward rounds, and used by the clinical team to ensure appropriate continuation of chronic medications in hospital. Patients are asked directly about their medication history, but when they are unable to provide details, the next of kin or general medical practitioner is contacted.

For the first 10 days of admission, we also collected physiological variables (systolic and diastolic blood pressure, heart and respiratory rates, temperature, oxygen saturation and neurological status), inflammation-related laboratory findings (white cell counts [WCC], and C-reactive protein [CRP]), and treatments received (oxygen supplementation, intravenous fluids, enteral nutrition, statins, other drugs). The blood tests described were not part of a study protocol and results were only available if these were collected as part of usual patient care. Follow-up continued until admission to HDU/ICU, discharge from hospital, or to a maximum of 10 days (including the day of admission), whichever came first. We also searched the hospital’s electronic patient record (EPR) to determine the patient’s vital status and location 30 days after admission (hospital ward; ICU/HDU; discharged from hospital). We recorded data related to re-admission to our hospital, if applicable.

Research assistants abstracted and recorded data on study forms, and two investigators (AD and MT) entered data into the study database (Microsoft© Access 2003, Microsoft Corporation, Seattle, USA), with each checking a random sample of data entered by the other for transcription errors. To further improve data integrity, the study database was programmed to reject out-of-range values, and an investigator (MT) checked a random sample of data recorded on study forms (approximately 10% of values or patients).

### Outcomes

Our primary outcome measures were the changes in the systemic inflammatory response syndrome (SIRS) during the follow-up period, defined as the proportion of patients who had at least one SIRS criterion on each day of follow-up, and the evolution of inflammation, as defined by trends in CRP and WCC. The criteria for SIRS included (1) temperature >38.3 or <36 degrees C, (2) respiratory rate >20 or PaCO_2_ < 32 mmHg, (3) heart rate >90 per minute, and (4) WCC >12 × 10^9^/L or <4 × 10^9^/L [1]. Because CRP and WCC had a skewed distribution, square root and logarithmic transformations were applied to these variables, respectively. Secondary outcomes of interest were mortality and admission to HDU/ICU. Vital status is reported as survival status either at hospital discharge or at 30 day follow up.

### Statistical analysis

We did not perform an *a priori* sample size calculation for this single-centre pilot observational study [16]; instead, we aimed to enrol all possible study subjects during the period of availability of the data collectors. Continuous data are expressed as means and standard deviations (SD) or medians and interquartile ranges (IQR), and categorical variables as frequencies and proportions.

Repeated measures linear regression models, which account for within-patient correlation, were used to determine associations between *a priori* selected predictors (days since admission, age, gender, Charlson score, prior statin exposure) and WCC and CRP. We used a generalized estimating equations model to determine associations between the same variables and the presence of any SIRS criteria on each day over the follow-up period. For all models, correlation was modelled using an autoregressive correlation structure. For the outcome of SIRS, we assumed that missing values represented normal values and that SIRS was present on the last day of recorded data for any patient who died or was transferred to HDU/ICU. For WCC and CRP, we assumed that values were missing at random. A two-sided p-value <0.05 was taken as statistically significant. Statistical analysis was performed using SAS version 9.3 (SAS Institute Inc., Cary, NC, USA).

## Results

### Description of cohort

We enrolled 209 patients over the study period (Table 1). Patients had a mean (SD) age of 63.8 (20.7) years and 104 (49.8%) were male. The commonest infection was pneumonia (n = 106, 51.2%), followed by urinary tract (n = 70, 34.1%) and skin/soft tissue infections (n = 38, 18.3%). Most patients (n = 184, 88.0%) had one or more SIRS criteria on day 1.

**Table 1:** **Baseline data in the study cohort**

Variable	Combined (n = 209)	Statin (n = 58)	Non-statin (n = 151)	p-value^1^
**Age (yr), mean ± SD**	63.8 ± 20.7	75.4 ± 11.3	59.4 ± 21.8	<0.0001
**Gender (male), n (%)**	104 (49.8)	27 (46.6)	77 (51)	0.57
**Charlson Index, median (IQR)**	2 (1–3)	3 (2–4)	1 (0–3)	<0.0001
**Type of infection** ^**2**^				
**Pneumonia**	106 (51.0)	27 (46.6)	79 (52.7)	0.43
**Primary bacteraemia**	6 (2.9)	2 (3.5)	4 (2.7)	0.68
**Peritonitis/intra-abdominal**	7 (3.4)	2 (3.5)	5 (3.4)	1.00
**Urinary tract**	70 (34.1)	23 (39.7)	47 (32.0)	0.30
**Skin/soft tissue**	38 (18.5)	12 (20.7)	26 (17.7)	0.69
**Other** ^**3**^	5 (2.7)	0 (0)	5 (3.3)	0.3252
**CRP (mg/L) (n = 176; 47; 129), median (IQR)**	65 (17–166)	87 (32–157)	58 (16–166)	0.51
**WCC (** **×10** ^**9**^ **/L) (n = 186; 48; 138), median (IQR)**	13.1 (9.1–17.7)	13.2 (9.4–17.9)	12.9 (9.0–17.6)	0.64
**Lowest systolic BP (mmHg) (n = 200; 54; 146), mean ± SD**	115 ± 22.1	119.4 ± 26.0	113.9 ± 20.3	0.12
**Highest temperature (Celsius) (n = 201; 55; 146), mean ± SD**	37.4 ± 0.99	37.4 ± 1.04	37.4 ± 0.97	0.70
**Lowest temperature (Celsius) (n = 199; 54; 145), mean ± SD**	36.5 ± 0.75	36.6 ± 0.80	36.4 ± 0.73	0.26
**At least 1 SIRS criterion, n (%)**	184 (88.0)	50 (86.2)	134 (88.7)	0.61
**Lowest SaO**_**2**_ **< 90%, n (%)**	22 (11.1)	8 (14.8)	14 (9.7)	0.30
**Supplemental oxygen (n, %)**	105 (50.5)	30 (51.7)	75 (50.0)	0.82
**Urine output (median [IQR]), (n = 40; 12; 28)**	750 (450–1237.5)	877.5 (417–1875)	750 (450–1237.5)	0.74
**AVPU (n, %) (n = 191; 54; 137)**				0.069
**Alert**	186 (97.4)	51 (94.4)	135 (98.5)	
**Responds to Voice**	4 (2.1)	3 (5.6)	1 (0.73)	
**Responds to Pain**	0 (0)	0 (0)	0 (0)	
**Unresponsive**	1 (0.5)	0 (0)	1 (0.73)	

Data refer to the first day of admission. Numbers in brackets, where provided, refer to the number of patients with data.

^1^For difference between statin versus non-statin groups.

^2^211 infections were identified. 4 patients did not have infection and 7 patients had >1 infection.

^3^Other infections included *C. difficile* (n = 1), osteomyelitis (n = 2), tonsillitis (n = 1), gastroenteritis (n = 1).

Fifty-eight patients (27.8%) satisfied the ‘statin user’ definition. Seven (4.6%) patients were given statin after admission in the previously no statin group and 54 (93.1%) were administered statin in the previously statin group. Among those receiving statin after admission, the percentage days of statin was 50 (33–60) in the non-statin and 90 (75–100) in the statin user group. Compared to non-statin users, patients on statins were older (75.4 ± 11.3 versus 59.4 ± 21.8 years, p < 0.0001) and suffered more chronic disease (Charlson score 3 [2–4] versus 1 [0–3], p < 0.0001), but there were no statistically significant differences between groups in terms of sex, markers of inflammation, or presence of at least 1 SIRS criteria on the first day [n = 50 (86.2%) vs. n = 134 (88.7%); p = 0.61].

### Outcomes and effect of statin exposure

Sixty-four (30.6%) patients deteriorated, as reflected by an increase in the number of positive SIRS criteria (Table 2). Progression of illness, defined by presence of more SIRS criteria than on study day 1, was more common in the statin group [47.4% vs. 33.3%; p = 0.063], although the difference was not statistically significant. The progression of illness as assessed using an increase in SIRS score over time were not different between the statin users and non-statin users among those whose SIRS score increased with time [median [IQR] days 2 (2–3) vs 2 (2–3) respectively; p = 0.57). There were no differences in evolution of other markers of organ dysfunction beyond day 1, including supplemental oxygen, urine output, and mental status. Seven patients (3.3%) required admission to HDU/ICU and twelve patients (5.7%) died within 30 days of admission. All the deaths were in a ward setting with one of the deaths following a readmission to hospital. The unadjusted hospital LOS was shorter in the non-statin group compared to the statin group [p = 0.013].

**Table 2:** **Outcomes in the cohort**

Outcome	Combined (n = 209)	Statin (n = 58)	Non-statin (n = 151)	p-value^1^
**Death by day 10 (n,%)**	5 (2.4)	2 (3.5)	3 (2.0)	0.62
**HDU/ICU admission (n,%)**	7 (3.3)	2 (3.4)	5 (3.3)	1.00
**HDU/ICU admission by day 10 (n,%)**	8 (3.8)	3 (5.2)	5 (3.3)	0.69
**Status at 30 days**				0.069
**Dead**	12 (5.7)	3 (5.2)	9 (6.0)	
**In ICU/HDU**	1 (0.48)	0 (0)	1 (0.66)	
**In ward**	27 (12.9)	13 (22.4)	14 (9.3)	
**Discharged from hospital**	169 (80.9)	42 (72.4)	127 (84.1)	
**More SIRS criteria at any time than recorded on day 1 (n,%)**	76 (37.3)	27 (47.4)	49 (33.3)	0.063
**Supplemental oxygen, days 2–10 (n,%)**	96 (47.1)	30 (52.6)	66 (44.9)	0.32
**Days on supplemental oxygen (median [IQR]), days 1–10**	1 (0–3)	1 (0–4)	1 (0–3)	0.28
**Days of antibiotics (median [IQR]), days 1–10**	5 (3–8)	6.5 (4–8)	5 (3–8)	0.10
**Urine output, days 2–10 (median [IQR] (n = 87; 27; 60)**	1125 (725–1701.4)	1110 (691–1635)	1157.9 (732.5–1764.6)	0.42
**Worst AVPU, days 2–10 (n,%) (n = 196; 54; 137)**				1.00
**Alert**	189 (96.4)	53 (96.4)	136 (96.5)	
**Responds to Voice**	7 (3.6)	2 (3.6)	5 (3.5)	
**Responds to Pain**	0	0	0	
**Unresponsive**	0	0	0	
**Hospital length of stay, days (median [IQR]** ^**1**^	8 (3–18)	10.5 (6–20)	7 (3–15)	0.013

Numbers in brackets, where provided, refer to the number of patients with data.

^1^ Hospital length of stay is calculated as the difference between discharge and admission date. If these dates are equal, it is counted as 1 day.

**Figure 1:**
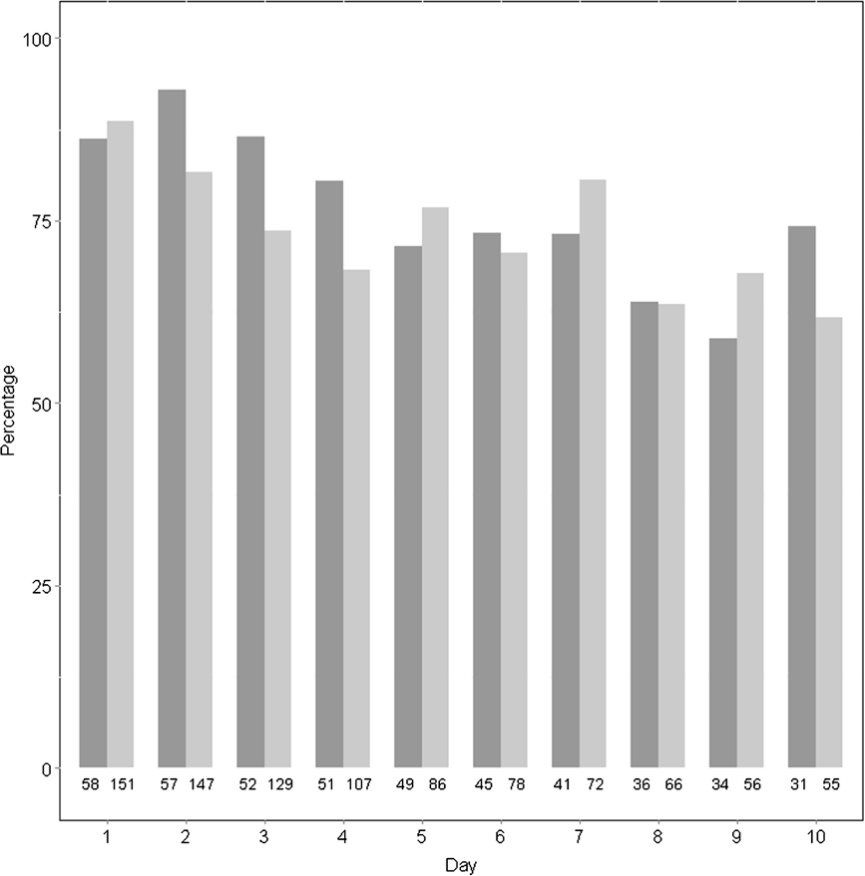
**Changes in SIRS criteria over time.** Percentage of patients with at least 1 SIRS criterion present on each day in statin users (black columns) vs. non-users (grey columns). Out of 341 patient-days coded as no SIRS there are 35 days for which values are missing for all variables in the SIRS criteria. The number of patients with data is below each column.

Overall, the proportion of patients with at least 1 SIRS criterion decreased with time in both statin users and non-users (Figure 1 and Table 3). When adjusted for other variables, the number of days since admission was significantly associated with the number of SIRS criteria positive (OR 0.86, 95% CI 0.82–0.91, p < 0.0001). None of age, sex, Charlson score, or statin exposure was associated with the presence of at least 1 SIRS criterion (Table 3). Out of 184 who had at least one SIRS criteria on day 1, 112 (60.9%) still had one SIRS on the last day of follow up. Of twenty five patients who did not have SIRS on day 1, 13 (52%) patients had new SIRS by the last day of follow up.

**Table 3:** **Adjusted model for associations with any SIRS criteria on any day of follow-up**

Variable	Odds ratio	95% Confidence interval	P value
**Days since admission**	0.86	0.82 - 0.90	<0.0001
**Age (per 10 year increase)**	1.06	0.96 - 1.17	0.27
**Gender (male versus female)**	1.10	0.74 - 1.62	0.64
**Charlson index (per 1 unit increase)**	1.05	0.97 - 1.13	0.24
**Statin (versus non-statin)**	1.13	0.76 - 1.69	0.55

WCC was missing on 614 (43.8%) of the 1401 patient-days of data. Using repeated measures analysis, the amount of missing data increases over time but does not differ between statin and non-statin groups.

**Figure 2:**
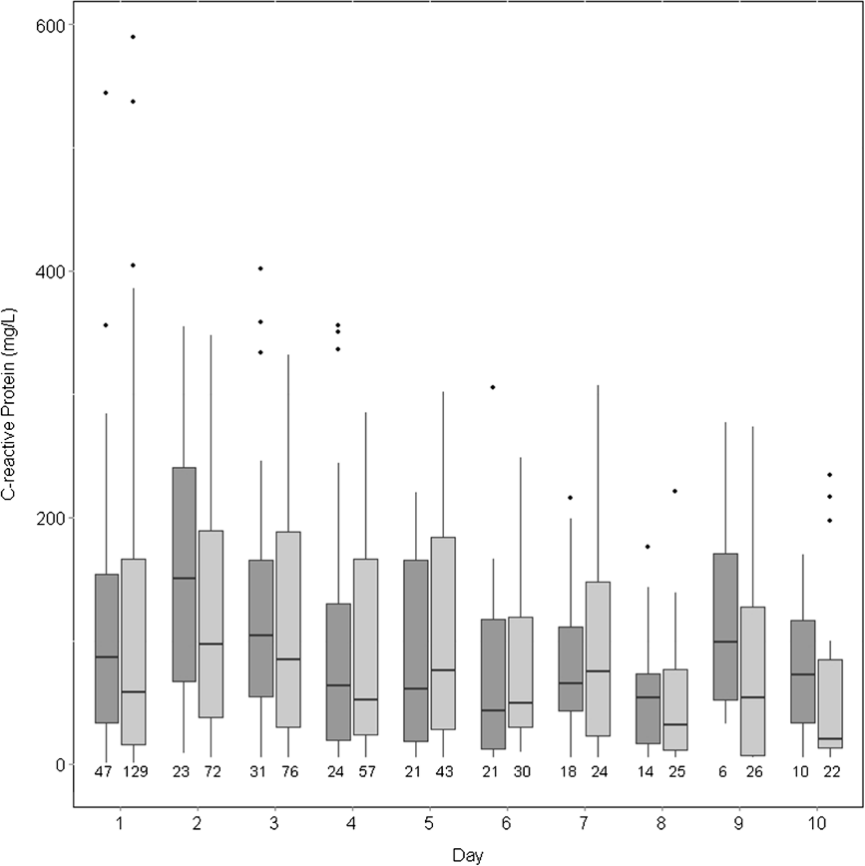
**Changes in C-reactive protein over the course of the study in statin users (dark grey) vs. non-users (light grey).** The number of patients with data is below each column. For each column, the dark horizontal line denotes the median value, the bottom and top of the box denote the first and third quartiles respectively, the upper whisker extends to the highest value that is within 1.5 × interquartile range of the third quartile, and the lower whisker extends to the lowest value within 1.5 × interquartile range of the first quartile. Data beyond the end of the whiskers are outliers and plotted as points.

**Figure 3:**
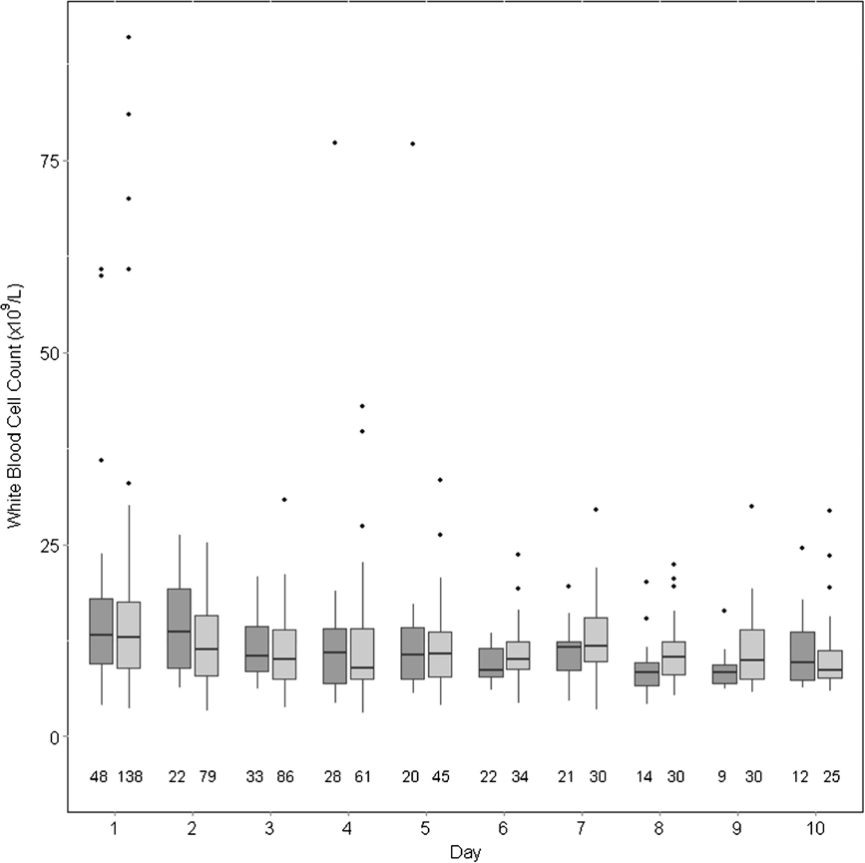
**Changes in white blood cell count over the course of the study in in statin users (dark grey) vs. non-users (light grey).** The number of patients with data is below each column. For each column, the dark horizontal line denotes the median value, the bottom and top of the box denote the first and third quartiles respectively, the upper whisker extends to the highest value that is within 1.5 × interquartile range of the third quartile, and the lower whisker extends to the lowest value within 1.5 × interquartile range of the first quartile. Data beyond the end of the whiskers are outliers and plotted as points.

CRP (Figure 2) and WCC (Figure 3) decreased over the follow-up period. When adjusted for other variables, the number of days since admission was again strongly associated with lower CRP (beta coefficient -0.3237 [standard error 0.065], p < 0.0001) and lower WCC (coefficient -0.0466 [standard error 0.007], p < 0.0001). None of age, sex, Charlson score, or statin exposure was associated with change in CRP or WCC (Tables 4 and 5).

**Table 4:** **Adjusted model for associations with square root of C-reactive protein**

Variable	Coefficient	Standard error	p value
**Days since admission**	-0.3237	0.066	<0.0001
**Age**	0.01337	0.016	0.3970
**Gender (male versus female)**	1.0358	0.600	0.0850
**Charlson index**	0.0369	0.139	0.7903
**Statin (versus non-statin)**	0.1063	0.693	0.8781

C-reactive protein was missing on 682 (48.7%) of the 1401 patient-days of data. Using repeated measures analysis, the amount of missing data increases over time but does not differ between statin and non-statin groups.

**Table 5:** **Adjusted model for associations with logarithm of white cell count**

Variable	Coefficient	Standard error	P value
**Time**	-0.0469	0.007	<0.0001
**Age**	0.0006	0.0015	0.6806
**Gender (male versus female)**	0.0599	0.057	0.2940
**Charlson index**	-0.0219	0.013	0.0952
**Statin (versus non-statin)**	0.037	0.066	0.5731

## Discussion

### Main findings

In our single-centre prospective cohort study of medical [non-surgical] patients admitted to hospital wards for treatment of suspected or confirmed infection, the risk of significant clinical deterioration was low, with a 30-day mortality of 5.7% and HDU/ICU admission risk of 3.3%. Markers of systemic inflammation (SIRS characteristics, CRP, WCC) improved with time. Prior statin therapy did not alter the trajectory of these inflammatory markers. Future larger studies may identify risk factors for progression of illness in this population and plausible surrogate endpoints for evaluation in clinical trials.

### Comparison to other work

Our main inclusion criterion, prescription of antibiotics for suspected or proven acute infection, was designed to identify a cohort for a future RCT of prevention of illness progression among non-surgical hospitalised patients with infection. This criterion was similar to the two published RCTs of statins for sepsis [12, 17]. One randomized trial of simvastatin for patients with bacterial infection, but not necessarily SIRS, found that only 4 of 83 patients progressed to severe sepsis [12]. More recently, another RCT of atorvastatin for hospitalised patients with acute infection and 2 SIRS criteria found that 14 of 100 patients progressed to severe sepsis, with a lower risk in the atorvastatin group (p = 0.007) [17]. The presence of SIRS is likely to be associated with the higher risk of progression of illness in the latter RCT, and may identify a higher risk target intervention group.

In our study cohort consisting of medical [non-surgical] patients, approximately one-third of patients had progression of illness defined by an increase in number of SIRS criteria. Compared to ward patients, the prevalence of SIRS in a critically ill population is high. ICU-based studies have evaluated the utility of SIRS progression either as a risk factor for more severe illness or as a risk factor for mortality [3, 5, 6, 18, 19]. Rangel-Frausto *et al* found a higher incidence of culture-positive sepsis with increase in number of SIRS criteria [3], but other studies have found that SIRS criteria have little value in detecting culture positive sepsis [20]. Similarly, patients with SIRS have a mortality rate of 10% [19] and while an increase in number of SIRS has been shown to be associated with an increase in risk of death [20], this relationship has not been replicated in other studies [6]. These inconsistent observations suggest that SIRS criteria lack specificity and sensitivity to predict progression of illness due to infection [2, 6, 19, 20]. Finally, the overall mortality in the study cohort was a limiting factor that precluded modelling the influence of SIRS criteria [2, 3, 5, 6], CRP trends [18, 21], or pneumonia [2, 22] on mortality.

### Strengths and limitations

Our study provides further insights into the natural history of bacterial infection following admission to a medical ward. We enrolled a clearly defined and general cohort and constructed multivariable models that accounted for within-patient correlation to explore the effect of pre-hospital statin exposure on markers of inflammation. Our study also has limitations. Many patients did not have CRP or WCC recorded on all days, presumably because they were clinically improving, possibly biasing association of statin with these outcomes to the null. The overall risk of clinically significant deterioration or death was low, precluding multivariable analyses of associations with these outcomes.

### Future directions

Patients with sepsis syndromes are clinically heterogeneous, with variable illness trajectory and outcomes. The utility of longitudinal trends in SIRS criteria and laboratory trends such as CRP to predict risk of progressive illness, ICU admission, and death must be further evaluated in a larger cohort of patients. The objective of such investigations would be to reliably identify a higher risk group of patients in whom strategies to prevent disease progression may be evaluated.

## Conclusions

We report a cohort of hospital admissions with bacterial infections requiring antibiotic therapy and assessed the trajectory of changes in SIRS criteria and inflammatory markers. The risk of clinically important adverse outcomes in this population was low, and time since admission was the only variable associated with trends in the number of SIRS criteria, CRP and WCC.

## Abbreviations

SIRS: Systemic inflammatory response syndrome
RCT: Randomised controlled trial
HDU: High dependency unit
ICU: Intensive Care Unit
CRP: C-reactive protein
EPR: Electronic patient records
WCC: White cell count.
